# Effects of dietary supplementation with *Bacillus subtilis* and bacteriophage on growth performance, intestinal morphology and microbiota structure in 0–90 d MaGang geese

**DOI:** 10.3389/fnut.2025.1537724

**Published:** 2025-04-02

**Authors:** Chenyu Zhu, Yongquan Luo, Kunjie Xu, Yong Li, Yuanhao Han, Zhiyuan Liu, Xiujin Li, Danning Xu, Yunbo Tian, Yunmao Huang, Zhongping Wu, Xumeng Zhang

**Affiliations:** Science and Technology Innovation Platform of Waterfowl Healthy Breeding in Guangdong, College of Animal Science and Technology, Zhongkai University of Agriculture and Engineering, Guangzhou, China

**Keywords:** goose, *Bacillus subtilis*, bacteriophages, growth performance, gut health

## Abstract

**Introduction:**

The expansion of large-scale goose farming under semi-arid conditions has exacerbated bathing pool pollution, adversely affecting goose growth performance and intestinal health. Given the crucial role of gut microbiota in maintaining intestinal homeostasis, and considering the reported beneficial effects of bacteriophages and *Bacillus subtilis* on gut health, this study investigated their combined application in goose production.

**Methods:**

To investigate the effects of *Bacillus subtilis* and phage supplementation on goose intestinal health, a 90-day trial was conducted with 288 Magang goslings randomly allocated to four treatment groups: control (A), *B. subtilis* (1 × 10^5^ CFU/kg; B), bacteriophage (5 × 10^7^ PFU/kg; C), and combined supplementation (D).

**Results:**

The supplementation significantly enhanced body weight (*p* < 0.05) and feed efficiency without affecting feed intake. Notably, the combined treatment demonstrated synergistic effects in reducing serum and aquatic endotoxin levels while suppressing pathogenic bacteria (*Escherichia coli* and *Salmonella*) in water systems. Intestinal morphology improvements included increased villus height and optimized villusto-crypt ratios, accompanied by up-regulated expression of tight junction genes (*Zo-1* and *Ocln*). Cecal microbiota analysis revealed enhanced alpha diversity and a shift toward Bacteroides-dominant communities, with concurrent suppression of Proteobacteria. Immune modulation exhibited a biphasic response, characterized by early anti-inflammatory (*Tnf-α*) and late-phase antioxidant (*Ho-1*) activities. *Microbialenvironmental* correlation analysis identified *Firmicutes* and *Desulfobacterota* as growth-promoting but barrier-compromising taxa, while *Bacteroidota* was associated with improved gut integrity.

**Conclusion:**

This research has shown that adding *B. subtilis* and bacteriophages to feed significantly enhances the intestinal barrier function of geese. The findings demonstrate that combined supplementation of *B. subtilis* and bacteriophages during the brooding and rearing stages optimizes growth performance through gut-microbiota-immune interactions, providing an effective antibiotic-free strategy for sustainable poultry production.

## 1 Introduction

Geese are one of the oldest commercially domesticated birds. However, advancements in waterfowl farming have led to significant consumption and pollution of water resources in traditional practices (1). Concurrently, the cleanliness of the water body affects the growth and development of animals and the ability to resist diseases to a certain extent (2, 3).

Lipopolysaccharide (LPS), a type of bacterial endotoxin found in the outer membrane of gram-negative bacteria, can contribute to water contamination and provoke inflammatory responses within the body. Over time, direct excretion into the water in geese can lead to increased levels of bacteria and LPS, which in turn leads to the accumulation of endotoxins in the geese’s body, which can result in disease and decreased performance (4).

Contamination of water bodies can lead to changes in the growth performance, intestinal structure and intestinal microbial composition of reared animals. The gut is a primary defense barrier against infection and is a key component of the animal’s immune system (5), and gastrointestinal microbes and their metabolites play a vital role in immune activation (6).

For waterfowl, the cleanliness of environmental water plays a critical role in modulating immune function. Conventional practices for managing bathing pool water in farming systems have been linked to adverse ecological impacts, including biological pollution and disease proliferation among waterfowl populations. Research by Yu et al. (4) underscores that high-density semi-dry farming practices exacerbate water pollution within farms. This compromises animal health and production performance. Such conditions have led to the overuse of antibiotics in goose farming, raising significant public health and environmental concerns (7). Consequently, there is an urgent need to explore feed additives as viable alternatives to antibiotics in goose production systems. The development of antibiotic-free farming practices could mitigate antimicrobial resistance risks while safeguarding food safety and ecological sustainability.

Probiotics play a critical role in balancing gut microbes and mitigating the effects of toxic metabolites, thereby improving gut health (8). Previous studies indicate that the performance of livestock is closely related to intestinal microbial load, the morphology and structure of the intestinal wall, and the activity of the immune system (9). *Bacillus subtilis* can serve as a non-toxic and non-residual probiotic product and it has been demonstrated that *B. subtilis* can maintain the balance of gut microbiota and the integrity of intestinal mucosal barrier, regulate nutrient metabolism, and strengthen animal immunity (10).

Bacteriophages are viruses that specifically target and utilize bacterial resources for their reproduction (11). Bacteriophages are specific for particular bacteria, and bacteriophage therapy is considered safe and effective in comparison to antibiotics partially because they infect one species, serotype or strain. This mechanism of action only inhibits the proliferation of the target microbial population (12, 13). In production practice, there are researches reported that *Salmonella* specific bacteriophages could serve as a potential method to reduce *Salmonella* in livestock (14, 15); previous studies by other researchers have shown that adding bacteriophages can reduce the colonization of *Salmonella* in the cecum of broiler chickens, and the addition of bacteriophages can reduce the colonization of *E. coli* in broiler chickens (14); Upadhaya et al. found that the inclusion of the 0.05% bacteriophage cocktail linearly improved broiler weight sufficient for supporting immune organs, bursa and spleen as well as enhancing gut microbiome (15).

At present, there are few reports on the combined application of bacteriophages and *B. subtilis* on the growth performance, bathing pool water, and cecal microbiota of MaGang geese. So, we fed 288 MaGang geese in four groups (group A, control group/basal diet; group B, *B. subtilis* group; group C, bacteriophage group; group D, mixed group). By tracking changes in goose growth performance, water environment, and cecal microbiota, we aim to uncover the impact mechanisms of bacteriophages and *B. subtilis* on goose production and cultivation. We found that the combination of bacteriophage and *B. subtilis* can effectively reduce environmental pollution, improve the intestinal microbial flora of geese and improve the production performance of geese. By improving the immunity of geese, we can reduce the mortality rate of goose farming, achieving the effect of replacing antibiotics.

## 2 Materials and methods

### 2.1 Animal ethics

This experiment was performed in accordance with the regulations and guidelines of the Animal Care Committee of the Zhongkai University of Agriculture and Engineering, and all efforts were made to minimize animal suffering. All experimental protocols were approved by the Animal Experiment Committee of Zhongkai University of Agriculture and Engineering (No. 2021112709).

### 2.2 Animals and materials

Healthy 1-day-old MaGang geese were obtained from Guangdong Lvfengyuan Modern Agricultural Development Co., Ltd. *B. subtilis* (≥1.0 × 10^8^ CFU/kg) was sourced from Huizhou Huinong Biotechnology Co., Ltd. (Guangdong, China). Bacteriophages (≥5.0 × 10^7^ CFU/mL) were purchased from Wuhan Greenong Biotechnology Co., Ltd. (Hubei, China), mainly containing a mixture of in a ratio of 1:1, targeting *Escherichia coli*, *Salmonella enterica*. The dosage of adding 0.1% bacteriophage or *B. subtilis* to the feed was based on pre-experiment results (Supplementary Figure S1) and previous studies (15–17).

### 2.3 Experimental design and diets

A total of 288 healthy MaGang geese (1 d) were randomly assigned to four experimental groups, with each group consisting of 6 replicate pens housing 12 geese each. The experimental groups were control group (group A, control group/basal diet), *B. subtilis* group (group B), bacteriophage group (group C) and mixed group (group D) (Table 1). The experiment was divided into 3 stages, the brooding period (1–20 days), the growing period (21–60 days), and the fattening period (61–90 days). On 20 d, all geese were shifted from the flat-raised mode in mesh pens to the semi-dry land farming mode, and the water in the bathing pool was changed every 10 days.

**Table 1:** Experimental group design.

Group	Treatment
A	Control, basal diet without treatment
B	Basal diet plus 1.0 × 10^8^ CFU/kg *Bacillus subtilis* at a concentration of 1/10^3^
C	Basal diet plus 5.0 × 10^10^ PFU/L bacteriophage at a concentration of 1/10^3^
D	Basal diet plus 5.0 × 10^10^ PFU/L bacteriophage at a concentration of 1/10^3^ and 1.0 × 10^8^ CFU/kg *Bacillus subtilis* at a concentration of 1/10^3^

Throughout the experimental period, all goslings were provided with water and a diet configured by the company at will, and the allowable error in the feed test results was referred to national standard (GB/18823). The feed is composed of corn, wheat, soybean meal, vegetable meal, sodium chloride, dicalcium phosphate, L-lysine sulfate, DL-methionine, vitamins and other components. Additives were added from the first day of gosling rearing (Supplementary Table S1).

### 2.4 Growth performance

Twenty-four geese (6 geese per group) were randomly selected and fasted for 12 h with access to water before slaughtering. Body weight of MaGang geese was measured every 10 days, feed consumption and weight gain were recorded during the experiment, average daily gain, average daily feed intake and feed-to-weight ratio were calculated.

### 2.5 Sampling procedure

The average body weight (BW), feed intake (FI) and feed conversion ratio (FCR) were calculated for the periods from 1 to 90 d, every 10 days. Geese were humanely euthanized via jugular vein exsanguination. On 20, 30, 60 and 90 d, the cecum and ileum was collected carefully in cryopreservation tubes. Sections of 1 cm were cut off from the middle of the ileum. The ileum sections were then fixed in 4% paraformaldehyde. The remaining length of the ileum was stored at −80°C for further analysis. Cecal chyme from geese at 20, 30, 60, and 90 days were collected and cryopreserved in liquid nitrogen for subsequent extraction of 16S rDNA. On 1, 20, 30, 60, 90 d, 6 geese were randomly selected from each group to collect their blood samples for serum separation for subsequent testing. On 30, 60, 90 d, water samples from the goose bathing pool were collected at the same location. Water samples were collected at a depth of 10–20 cm in the four corners and central part of the bathing pool, and the points were mixed as a single sample.

### 2.6 Endotoxin content

Horseshoe crab reagent (BIOENDO, Fujian) was used to detect endotoxins in serum on 1, 20, 30, 60, and 90 d, as well as in water samples on 30, 60, and 90 d.

First, the standard endotoxin solution was accurately diluted to create a series of standard points with varying concentrations. Then, the standard points of each concentration were combined with the horseshoe crab reagents in the proportions specified in the instructions. The mixture was incubated at a constant temperature (typically 37°C) for a designated time (10–15 min), and the absorbance was measured using a microplate reader. When plotting the standard curve, the standard endotoxin concentration was used as the abscissa, while the corresponding absorbance served as the ordinate. A set of controls containing only horseshoe crab reagent and buffer, without the sample to be tested, was incubated, and absorbance was determined under the same conditions. Depending on the nature of the sample and the anticipated endotoxin concentration, the appropriate dilution factor was chosen to dilute the sample for testing. The diluted sample was mixed with horseshoe crab reagent according to the instructions. Subsequently, the mixture of the sample and horseshoe crab reagent was placed in a thermostatic device and maintained at a constant temperature for the incubation time recommended by the instructions (usually the same as that for standard curve preparation). After incubation, the mixture was promptly transferred to a microplate reader, and the absorbance was measured at the same wavelength as the standard curve. The absorbance value of the sample to be measured was converted to the corresponding endotoxin concentration using the standard curve (the result should be adjusted by subtracting the absorbance background of the blank control).

### 2.7 Bacterial colony culture

Preparation of plate counting agar (PCA) medium (produced by Qingdao High-tech Industrial Park Haibo Biotechnology Co., Ltd): the international standard plate counting agar, containing sugar, is used for the determination of the total number of bacteria.

Preparation of MacConkey agar medium (produced by Guangdong Huankai Microbial Sci. & Tech. Co., Ltd.): this medium is widely used in the selective enrichment culture of *E. coli* for the detection and verification of coliform titers in water, milk and other foods, which presents pink colonies on plates.

Preparation of SS agar medium (produced by Guangdong Huankai Microbial Sci. & Tech. Co., Ltd.): this medium is mainly used for the isolation culture of *Salmonella*. Most *Salmonella* do not ferment lactose and produce hydrogen sulfide, and the colonies on SS plates are colorless and transparent with black centers, while *Salmonella* that do not produce hydrogen sulfide has no black centers.

Live bacterial count: water samples were sterilized on an ultra-clean bench and appropriately diluted to a dilution factor of 10^3^ or 10^4^. For the coating culture, 100 μL of the diluted sample was taken, and a coating rod was used to ensure even distribution of the bacterial solution. Subsequently, during the plating culture phase, disposable culture dishes were laid flat in the incubator for 10–20 min before being incubated at 37°C for 18–24 h. After incubation, the counting statistics were performed on the colonies present on the nutrient agar plates, the red colonies on the MacConkey agar plates, and the black colonies on the SS agar plates; colonies were counted between 30 and 300. Finally, the average number of colonies from the three replicate plates of the same dilution gradient was calculated, allowing for the determination of the number of viable bacteria per milliliter of the solution.

### 2.8 Sample collection and H&E

After fixation in 4% paraformaldehyde for 24 h, the ileum sections were dehydrated through a series of graded ethanol and xylene and then embedded in paraffin. Transverse 5 μm sections were stained with hematoxylin and eosin. Histomorphological parameters were examined using the micrographs taken with the Axio Imager Z1 (ZEISS Germany), zooming in 400× for each area and analyzed with CaseViewer 2.2 scanning and viewing software. The villus height and crypt depth of the slice samples were measured, and the ratio of villus height to crypt depth was calculated. For each slice sample, 10 complete and vertical villi were selected. Villus height was measured from the top of the villus to the crypt opening, and crypt depth was measured from the crypt opening to the base of the crypt. Histomorphological measurements of villi were performed using CaseViewer 2.2 software. One-way ANOVA was performed for different groups at each period, using the control group as the baseline.

### 2.9 RNA extraction and real-time quantitative PCR

The ileum samples were collected and immediately placed in RNase-free centrifuge tubes, followed by rapid freezing in liquid nitrogen. Total RNA isolation was performed using 100 mg tissue samples and 1 mL Trizol reagent (Thermo Fisher, Waltham, MA, USA). After extraction, the total RNA was dissolved in DEPC water, and the purity and concentration of RNA were measured with full wavelength spectrophotometer. The reverse transcription experiment was carried out in accordance with the reverse transcription kit ReverTra Ace qPCR RT Master Mix with gDNA Remover (TOYOBO, Osaka, Japan) instructions. By comparing with GenBank in the NCBI database, real-time PCR primers were designed by software Primer Premier (Version 5.0) (Table 2). In this study, *β-actin* was used as internal reference gene to detect gene expression by RT-qPCR. The cDNA was used according to the instructions of SYBR Select Master Mix kit (Thermo, USA), 20 μL reaction system was used and prepared on ice. The relative mRNA expression was calculated by the 2^−∆∆CT^ method, and the Tukey method was used to compare the experimental groups in the same period.

**Table 2:** Statistical analysis of growth performance.

Items	Groups				*η* ^2^	95% Confidence Interval of the Mean	
	A	B	C	D		Lower limit	Upper limit
BW, g							
Day 10	382.23 ± 41.54^**c**^	405.60 ± 33.08^**b**^	433.17 ± 40.84^**a**^	420.97 ± 37.50^**ab**^	0.20	402.82	418.16
Day 20	926.60 ± 94.21^**c**^	949.53 ± 78.33^**bc**^	994.93 ± 68.93^**a**^	987.47 ± 68.55^**ab**^	0.12	949.79	979.48
Day 30	1,398.37 ± 184.39^**b**^	1,460.17 ± 152.78^**ab**^	1,526.20 ± 209.39^**a**^	1,469.67 ± 138.30^**ab**^	0.07	1,431.57	1,495.63
Day 40	1,902.50 ± 150.50^**c**^	2,045.00 ± 143.20^**b**^	2,198.50 ± 127.90^**a**^	2,136.70 ± 100.90^**ab**^	0.20	2,144.26	2,226.06
Day 50	2,415.33 ± 298.07^**c**^	2,607.40 ± 261.04^**b**^	2,791.33 ± 261.91^**a**^	2,741.33 ± 209.56^**a**^	0.25	2,585.52	2,692.18
Day 60	2,848.40 ± 362.10^c^	3,123.10 ± 258.80^a^	3,272.90 ± 348.60^a^	3,048.30 ± 305.60^b^	0.19	3,009.49	3,136.91
Day 70	2,960.03 ± 301.46^b^	3,310.33 ± 286.16^a^	3,454.13 ± 265.35^a^	3,352.03 ± 255.23^a^	0.32	3,209.18	3,329.09
Day 80	3,300.70 ± 287.30^b^	3,560.30 ± 312.90^a^	3,611.00 ± 380.60^a^	3,365.20 ± 362.20^b^	0.13	3,394.59	3,524.02
Day 90	3,245.17 ± 344.74^c^	3,644.60 ± 262.68^a^	3,716.80 ± 397.93^a^	3,430.37 ± 365.63^b^	0.23	3,438.86	3,579.61
ADFI, g							
1–10 days	38.99 ± 15.60	41.10 ± 15.73	40.47 ± 15.58	39.42 ± 14.31	0.05	35.03	44.96
11–20 days	97.38 ± 11.47	97.91 ± 11.06	98.69 ± 11.45	94.39 ± 9.43	0.02	93.71	100.48
21–30 days	136.87 ± 31.17	144.20 ± 31.77	147.22 ± 30.71	135.54 ± 30.26	0.03	131.31	150.61
31–40 days	194.32 ± 14.43	189.87 ± 19.46	199.77 ± 20.27	188.32 ± 15.89	0.07	187.45	198.69
41–50 days	209.55 ± 16.14	210.97 ± 16.80	219.59 ± 15.90	222.13 ± 25.44	0.08	209.47	221.65
51–60 days	209.88 ± 25.67	215.89 ± 26.66	224.38 ± 26.93	207.90 ± 25.64	0.06	206.19	222.83
61–70 days	214.13 ± 19.06	212.93 ± 20.21	202.61 ± 18.20	201.74 ± 24.31	0.08	201.27	214.44
71–80 days	186.92 ± 10.83	201.25 ± 45.57	198.12 ± 28.58	188.58 ± 22.75	0.05	184.38	203.05
81–90 days	188.17 ± 28.45	207.35 ± 22.61	212.53 ± 16.62	207.11 ± 32.76	0.13	195.30	212.28
FGR							
1–10 days	1.44 ± 0.57	1.37 ± 0.53	1.26 ± 0.49	1.27 ± 0.46	0.02	1.17	1.50
11–20 days	1.79 ± 0.21	1.80 ± 0.20	1.76 ± 0.20	1.66 ± 0.16	0.10	1.68	1.81
21–30 days	2.90 ± 0.66	2.82 ± 0.62	2.77 ± 0.58	2.86 ± 0.64	0.01	2.65	3.03
31–40 days	3.05 ± 0.23^**a**^	2.72 ± 0.28^**b**^	2.55 ± 0.26^**bc**^	2.46 ± 0.20^**c**^	0.49	2.59	2.80
41–50 days	4.09 ± 0.31^a^	3.75 ± 0.30^b^	3.70 ± 0.27^b^	3.67 ± 0.42^b^	0.22	3.69	3.92
51–60 days	4.85 ± 0.59^b^	4.19 ± 0.52^c^	4.66 ± 0.56^b^	5.03 ± 0.62^a^	0.25	4.48	4.88
61–70 days	18.42 ± 1.56^a^	11.58 ± 0.80^b^	11.40 ± 0.65^b^	6.82 ± 0.72^c^	0.93	9.82	12.50
71–80 days	4.98 ± 0.24^d^	7.09 ± 0.54^c^	10.22 ± 0.54^b^	22.46 ± 1.38^a^	0.97	6.90	10.74
81–90 days	6.05 ± 1.11^b^	5.67 ± 0.80^b^	6.85 ± 1.04^b^	13.57 ± 3.13^a^	0.80	6.29	8.71

BW, Body weight; ADFI, Average Daily Feed Intake; FGR, Feed to Gain Ratio (*n* = 6); ^a,b,c,d^Means in the same row with different superscripts (a, b, c, d) differ significantly between columns (*p* < 0.05).

### 2.10 16S rDNA sequencing of cecal bacteria

#### 2.10.1 Sub-region sequencing (Illumina)

**DNA extraction**: Microbial DNA was extracted using the HiPure Soil DNA Kits (or HiPure Stool DNA Kits) (Magen, Guangzhou, China) according to manufacturer’s protocols.

**PCR amplification**: The 16S rRNA target region of the ribosomal RNA gene was amplified by PCR (95°C for 5 min, followed by 30 cycles at 95°C for 1 min, 60°C for 1 min, and 72°C for 1 min and a final extension at 72°C for 7 min) using primers listed in Supplementary Table S1. 50 μL of the mixture includes 10 μL of 5 × Q5@ Reaction Buffer, 10 μL of 5 × Q5@ High GC Enhancer, 1.5 μL of 2.5 mM dNTPs, 1.5 μL of each primer (10 μM), 0.2 μL of Q5@ High-Fidelity DNA Polymerase, and 50 ng of template DNA. The related PCR reagents were sourced from New England Biolabs, USA.

**Illumina Novaseq 6000 sequencing**: Amplicons were evaluated with 2% agarose gels and purified using the AMPure XP Beads (Beckman, CA, USA) according to the manufacturer’s instructions. Sequencing libraries were generated using Illumina DNA Prep Kit (Illumina, CA, USA) following manufacturer’s recommendation. The library quality was assessed with ABI StepOnePlus Real-Time PCR System (Life Technologies, Foster City, USA). At the end 2 × 250 bp paired-end reads were generated by sequencing on the Novaseq 6000 platform. Guangzhou Gene Denovo Biotechnology Co., Ltd. conducted the sequencing analysis.

**Analysis software**: FASTP (version 0.18.0) was used to filter raw data from the Illumina platform, OTU stands for sequence or ASV sequence alignment SILVA database (version 138.1) or UNITE database (version 8.3) was used for species classification annotation using the naïve Bayes model of RDP annotation software (version 2.2) with a confidence threshold of 0.8–1. Use Krona (version 2.6) to display abundance statistics for each species taxonomy. Venn analysis using the R VennDiagram package (version 1.6.16) and the R language UpSetR package (version 1.3.3) was analyzed for endemic species, OTU, ASV between groups. Chao1, ACE, Shannon, Simpson, Good’s coverage, Pielou’s evenness diversity index is calculated in QIIME (version 1.9.1). OTU representative sequence alignment was performed using Muscle (version 3.8.31), phylogenetic trees were constructed using FastTree (version 2.1), and then weighted and unweighted unifrac distance matrices were generated using the R language GuniFrac package (version 1.0). Moreover, the correlation index was calculated by Spearman’s correlation method using the OmicShare tools.^1^

### 2.11 Statistical analysis

Six biological replicates were set in each group. One-way analysis of variance (ANOVA) was conducted using Graphpad Prism software (Version 5.0), and difference significance analysis was performed using Tukey’s test. Correlation analysis uses Pearson correlation coefficients for descriptive statistics. *p* > 0.05 means that the difference is not significant, while *p* < 0.05 means that the difference is significant.

## 3 Results

### 3.1 Effects of *Bacillus subtilis* and bacteriophages on growth performance

We conducted a statistical analysis of the average body weight (BW), average daily feed intake (ADFI), and average feed-to-gain ratio (FGR) for each group at various stages of the study (Table 2). Throughout the experimental period, group A (control group) consistently exhibited lower values than the other three experimental groups. By days 10 and 20, the average body weights of group B (*B. subtilis* group), group C (bacteriophage group), and group D (mixed group) were significantly higher than that of group A (*p* < 0.05). Notably, group C exhibited the highest body weight and showed significantly higher than group B. By day 30, group C exhibited the highest BW, which was significantly higher than that of group A (*p* < 0.05). From days 40 to 60, the weights of groups B, C, and D were significantly higher than that of group A (*p* < 0.05). By day 40, group C exhibited the highest weight and significantly higher than group B. However, significant differences emerged between groups B and D by days 50 and 60 (*p* < 0.05). Specifically, group C was significantly higher than group B (*p* < 0.05), and although group D was higher than group B, this difference was not statistically significant. From days 70 to 90, the weights of groups B, C, and D remained significantly higher than that of group A (*p* < 0.05). Nevertheless, the weight of group D was significantly lower than that of groups B and C, except by day 70.

The ADFI results showed that there were no significant differences (*p* > 0.05) between the four groups throughout the experimental period, which indicated that *B. subtilis* and bacteriophages did not affect the normal feeding intake of geese. For FGR, no significant differences were observed among the four groups during the 1–30 d period. However, from days 31 to 50, the FGR of groups B, C, and D were significantly lower than that of group A (*p* < 0.05), indicating that diets containing *B. subtilis* or bacteriophages during the incubation stage offered higher economic benefits. From days 51 to 60, groups B and D were significantly lower than that of group A (*p* < 0.05). During the 61–70 d period, group D had significantly lower ratio than those of the other three groups (*p* < 0.05), with group A exhibiting the highest. However, from days 71 to 90, group D had significantly higher ratio than those of the other three groups (*p* < 0.05). In contrast, group A showed lower levels among the groups during days 71–90.

These findings suggest that diets incorporating both *B. subtilis* and bacteriophages provided the greatest economic advantages during 1–70 d period. Overall, these results indicate that the incorporation of *B. subtilis* and bacteriophages in the brooding and rearing stages can effectively enhance the growth performance of geese and reduce breeding costs.

### 3.2 Effects of dietary supplementation with *Bacillus subtilis* and bacteriophages on the total bacteria on PCA plates colonies in cultured water

To investigate the effects of *B. subtilis* and bacteriophages on goose bathing pool water, this study initially simulated bathing conditions using goose fecal water to determine the optimal concentrations of bacteriophages and *B. subtilis* under simulated conditions (Supplementary Figures S1A,B). In addition, endotoxin levels, bacterial levels in the water (Supplementary Figure S1C), and total number of colonies were measured to ensure that these treatments did not interfere with each other and did not affect the physicochemical properties of the water (Supplementary Figure S2).

**Figure 1:**
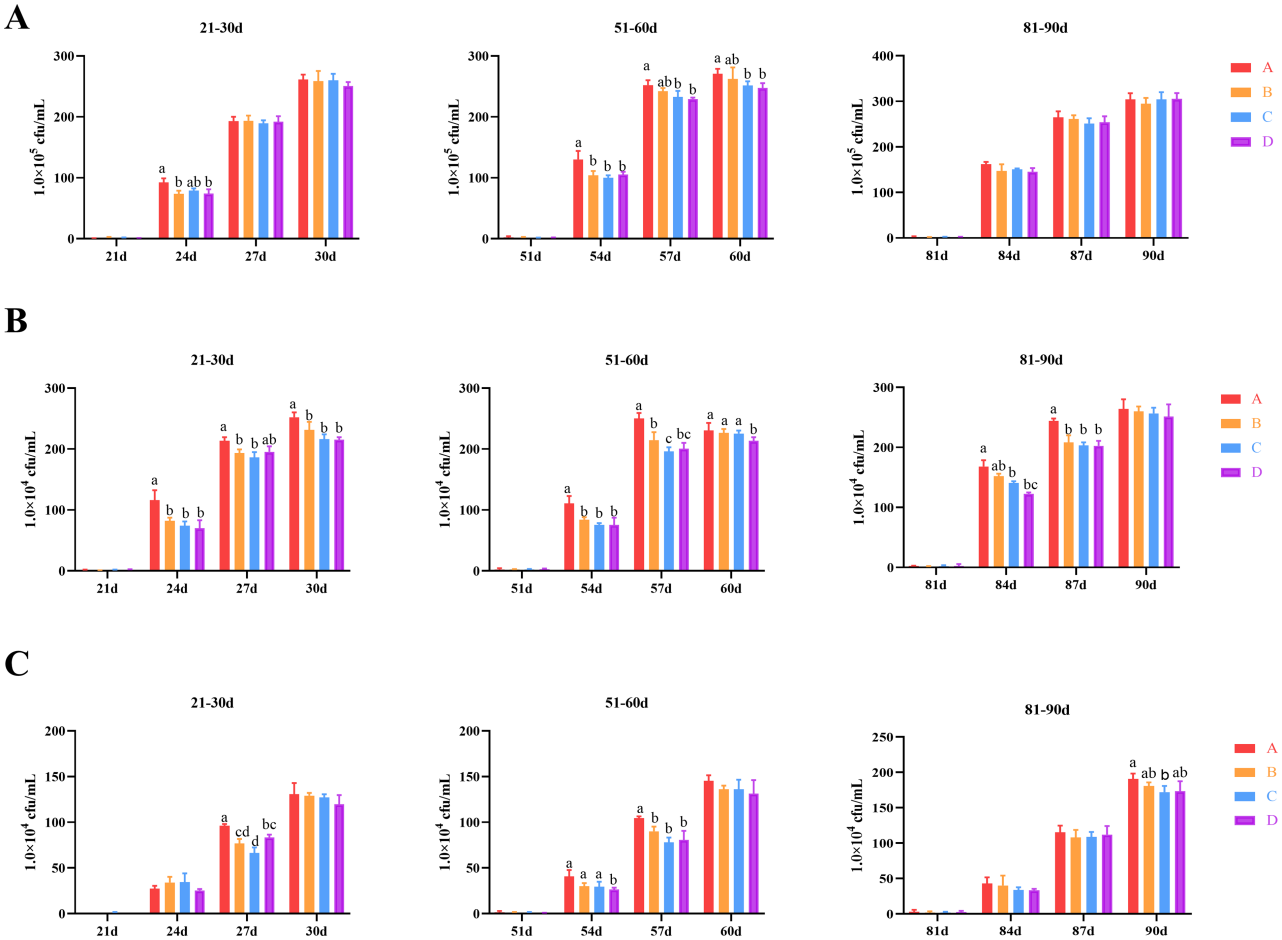
Effects of dietary supplementation of *Bacillus subtilis* and bacteriophages on bacteria in cultured water. **(A)** Total colony counts in cultured water (total bacteria on PCA plates). **(B)** *E. coli* counts in cultured water. **(C)** *Salmonella* counts in cultured water. ^a,b^Means with different superscripts (a, b) between two columns are significantly different (*p* < 0.05), the same below.

In this study, we investigated the impact of bacteriophages and *B. subtilis* on the bacterial populations in the bathing pool water of MaGang geese at various developmental stages over a 10-day period following water replacement. Our findings revealed that the total bacteria on PCA plates colonies exhibited an increasing trend over time. Specifically, from days 21 to 30, we observed the total number of colonies in groups B, C and D exhibited a decrease compared to group A by day 24, 3 days after the water replacement. However, only groups B and D demonstrated statistically significant differences from group A (*p* < 0.05). By days 27 and 30, there were no significant differences in the number of colonies among all groups. From days 51 to 60, the total number of colonies in groups B, C and D showed a significant reduction compared to group A (*p* < 0.05). By days 57 and 60, the total number of colonies in groups B, C and D continued to decreased compared to group A, with statistically significant differences only observed between groups B and D when compared to group A (*p* < 0.05) (Figure 1A).

Regarding *Escherichia coli* (*E. coli*) levels, the overall trend was consistent with the total number of colonies observed. In the period from days 21 to 30, 3 days after changing the water, groups B, C and D exhibited a significant reduction compared to group A (*p* < 0.05), with the exception of group D by day 27. From days 51 to 60, the *E. coli* levels in groups B, C and D showed a significant reduction compared to group A (*p* < 0.05) by day 54. By day 57, groups B, C and D once again exhibited a significant reduction compared to group A, and additionally, the *E. coli* levels in group C were significantly lower than those in group B (*p* < 0.05). However, by day 60, only group D had significantly lower *E. coli* levels compared to group A (*p* < 0.05), with no significant differences observed between groups B and C and group A. By day 84, a gradually decreasing trend in *E. coli* levels was observed among all groups. Specifically, groups C and D had significantly lower *E. coli* levels compared to group A (*p* < 0.05) (Figure 1B).

The overall trend in *Salmonella* levels was also consistent with the total number of colonies observed. From days 21 to 30, significant differences were observed among all groups by day 27 specifically. Groups B, C, and D exhibited significantly lower *Salmonella* levels compared to group A (*p* < 0.05). Furthermore, group C showed significantly lower levels compared to group D (*p* < 0.05). During the period from days 51 to 60, significant differences were observed between group D to groups A, B and C (*p* < 0.05). Notably, groups B, C, and D continued to exhibit significantly lower *Salmonella* levels compared to group A (*p* < 0.05). From days 81 to 90, only group C showed significantly lower *Salmonella* levels compared to group A at day 90 (*p* < 0.05) (Figure 1C).

Collectively, these results suggest that the application of bacteriophages and *B. subtilis* can significantly reduce the levels of total colonies, *E. coli*, and *Salmonella* in bathing pool water for the first 6 days following a water change.

### 3.3 Effects of dietary supplementation with *Bacillus subtilis* and bacteriophages on serum and cultured water endotoxin content

**Figure 2:**
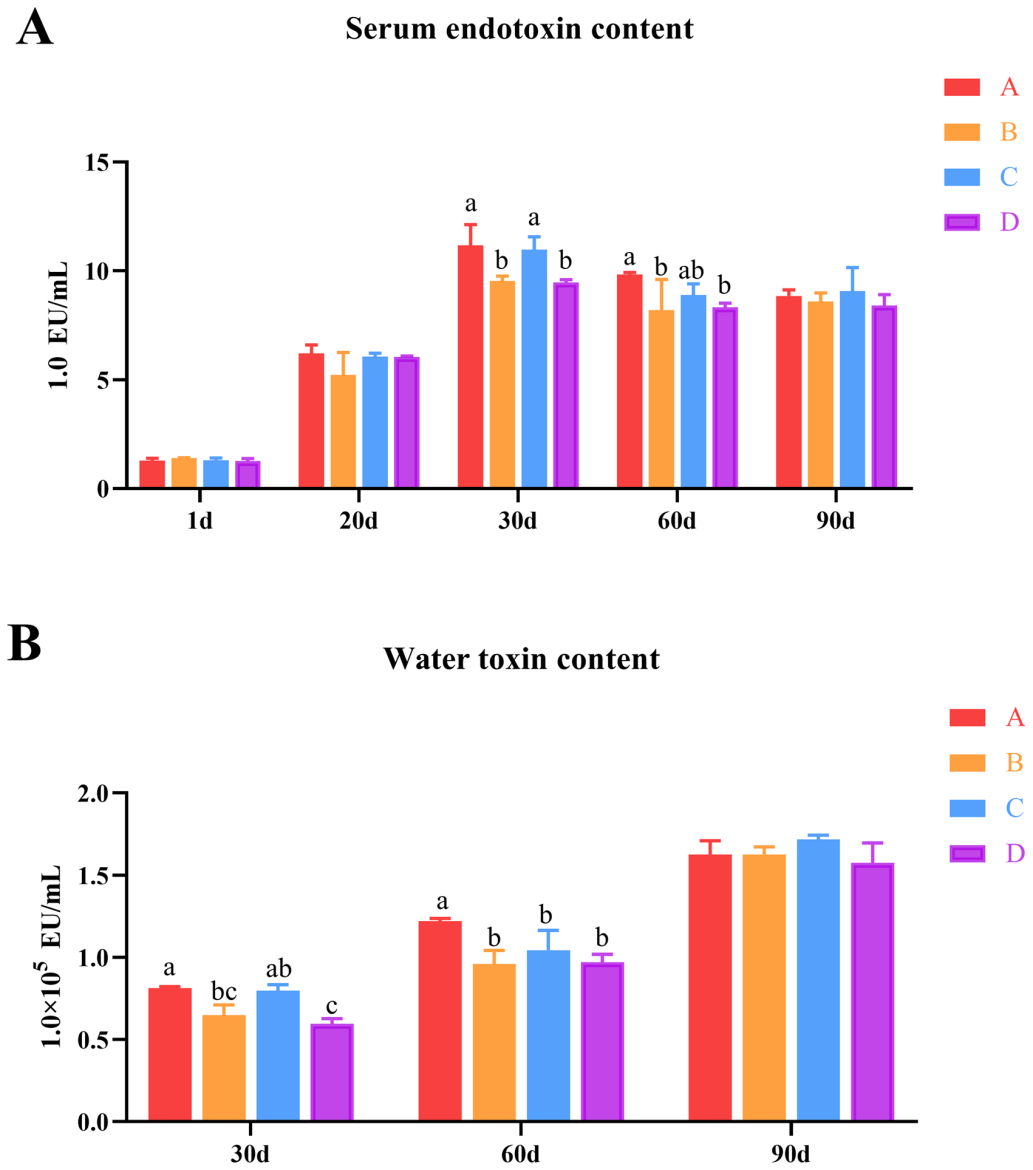
Effects of dietary supplementation of *Bacillus subtilis* and bacteriophages on serum and water endotoxin levels in cultured water. **(A)** Serum endotoxin levels in geese. **(B)** Water endotoxin levels in cultured water. ^a,b^Means with different superscripts (a, b) between two columns are significantly different (*p* < 0.05), the same below.

To investigate the effects of *B. subtilis* and bacteriophage on the farmed water and serum endotoxin levels in MaGang geese, this study employed the horseshoe crab reagent colorimetric methods to analyze bath water and serum endotoxin levels. The results indicated that the serum endotoxin levels of MaGang geese increased over time and stabilized after 30 days. By days 30 and 60, groups B and D exhibited significant reductions in serum endotoxin levels, whereas group C showed no significant change compared to group A. By day 90, the endotoxin content in the serum of group C was the highest, however, there were no significant differences among the groups (*p* > 0.05) (Figure 2A).

The endotoxin levels in bathing pool water gradually increased over time. By day 30, groups B and D showed significantly reduction of the endotoxin levels in the water, while group C showed no significant change compared to group A. There was no significant difference in the comparison between group B and group C. However, group D exhibited significantly lower levels compared to group C. Notably, there was no significant difference observed between group B and group D. By day 60, groups B, C and D exhibited significantly reduced the endotoxin content in water compared to group A (*p* < 0.05), while there were no significant differences among groups B, C and D (*p* > 0.05) (Figure 2B). These results indicate that the addition of microecological preparations resulted in a reduction of endotoxin levels in both the water and the geese’s serum from days 30 to 60, with group B and D exhibiting the most significant effects (*p* < 0.05).

### 3.4 Effects of dietary supplementation with *Bacillus subtilis* and bacteriophages on intestinal morphology

**Figure 3:**
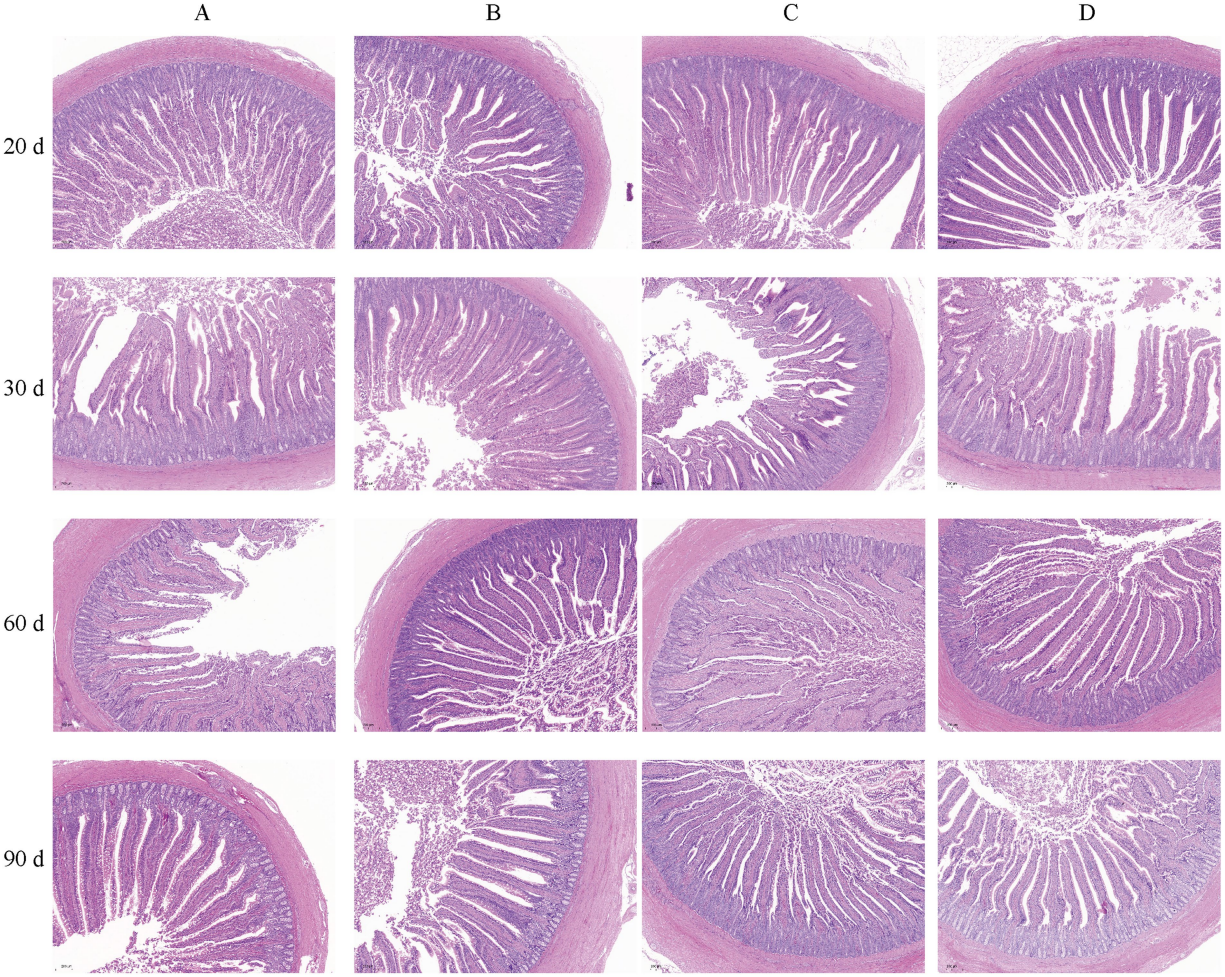
Morphology of the iejunum in MaGang geese treated with bacteriophage and *Bacillus subtilis* (×200). Scale bar = 200 μm.

To investigate the effects of bacteriophages and *B. subtilis* on the health of the jejunum of MaGang geese, we analyzed histological sections of the jejunum, focusing on villus height, crypt depth, and jejunal villus-to-crypt ratio (Figure 3 and Table 3).

**Table 3:** Statistical analysis of intestinal histology.

Item type		VH (μm)					CD (μm)					VH/CD				
		Day 1	Day 20	Day 30	Day 60	Day 90	Day 1	Day 20	Day 30	Day 60	Day 90	Day 1	Day 20	Day 30	Day 60	Day 90
Group A		249.56 ± 42.8^b^	741.30 ± 100.66^b^	1,067.97 ± 33.51^ab^	869.7 ± 42.18^b^	912.09 ± 65.58^ab^	104.16 ± 9.27^b^	273.33 ± 32.52	362.27 ± 53.21^a^	244.93 ± 6.1	209.36 ± 21.95^ab^	2.39 ± 0.34	2.77 ± 0.74^ab^	3.00 ± 0.53^c^	3.56 ± 0.26^b^	4.38 ± 0.3^ab^
Group B		164.10 ± 12.23^c^	707.30 ± 23.66^b^	953.4 ± 54.2^b^	1,019.93 ± 76.08^a^	939.9 ± 87.02^a^	76.07 ± 14.64^c^	242.27 ± 6.28	189.07 ± 12.91^c^	237.27 ± 21.47	221.16 ± 22.64^b^	2.20 ± 0.31	2.92 ± 0.17^b^	5.05 ± 0.15^a^	4.34 ± 0.68^ab^	4.28 ± 0.55^ab^
Group C		262.50 ± 2.26^b^	1,011.50 ± 88.46^a^	1,200.47 ± 120.94^a^	1,003.53 ± 75.67^a^	948.31 ± 133.14^a^	114.97 ± 11.65^ab^	264.03 ± 9.15	241.27 ± 10.2^bc^	241.5 ± 38.47	250.41 ± 20.47^a^	2.30 ± 0.24	3.83 ± 0.29^a^	4.97 ± 0.29^a^	4.23 ± 0.71^ab^	3.79 ± 0.44^c^
Group D		354.8 ± 18.45^a^	956.43 ± 34.52^a^	1,045.83 ± 48.53^b^	1,118.93 ± 35.63^a^	826.56 ± 69.89^b^	139.73 ± 19.44^a^	244.97 ± 17.43	285.47 ± 3.04^b^	223.93 ± 21.26	186.19 ± 18.95^c^	2.57 ± 0.37	3.92 ± 0.35^b^	3.66 ± 0.16^b^	5.02 ± 0.36^a^	4.50 ± 0.8^a^
*p* value	B–A	0.045	0.931	0.287	0.062	0.943	0.112	0.273	<0.001	0.980	0.723	0.973	0.974	<0.001	0.343	0.989
	C–A	0.956	0.007	0.193	0.099	0.884	0.908	0.932	0.03	0.998	0.007	1.000	0.074	<0.001	0.464	0.222
	D–A	0.016	0.023	0.981	0.004	0.334	0.094	0.338	0.039	0.730	0.195	0.775	0.052	0.128	0.041	0.973
*η* ^2^		0.87	0.84	0.69	0.76	0.61	0.79	0.41	0.89	0.14	0.58	0.21	0.67	0.92	0.58	0.55
95% Confidence Interval of the Mean		(312.362, 397.238)	(862.976, 1,049.891)	(949.268, 1,142.398)	(1,038.599, 1,199.267)	(754.126, 898.988)	(120.465, 159.002)	(219.315, 270.619)	(248.333, 322.600)	(191.117, 256.750)	(169.764, 202.607)	(2.127, 3.016)	(3.329, 4.511)	(3.236, 4.091)	(4.305, 5.733)	(4.072, 4.936)

VH, Villus Height; CD, Crypt Depth; VH/CD, Villus Height relative to Crypt Depth (*n* = 6); ^a,b,c^Means in the same row with different superscripts (a, b, c) differ significantly between columns (*p* < 0.05).

Our findings revealed that by day 20, groups C and D exhibited significantly villus heights compared to groups A and B (*p* < 0.05). However, there were no significant differences in villus height between groups A and B, or between groups C and D (*p* > 0.05). By day 30, group C had a significant higher villus height compared to groups B and D (*p* < 0.05). By day 60, significant differences were observed between groups B, C, and D compared to group A (*p* < 0.05). By day 90, groups B and C exhibited significantly higher villus heights than group D (*p* < 0.05), while no significant differences were observed compared to group A (*p* > 0.05).

In terms of crypt depth, by day 30, group A had the highest crypt depth, significantly differing from the other three groups, and group D had a significant higher crypt depth compared to group B (*p* < 0.05). By day 90, group D exhibited significantly lower crypt depth than the other three groups, and group B had significant lower than group C.

Regarding the jejunal villus to crypt ratio, by day 20, groups B and D exhibited significantly higher ratios than group C (*p* < 0.05), while no significant differences were observed compared to group A (*p* > 0.05). By day 30, group A exhibited significantly lower ratios than the other three groups, and group D had a significant lower ratio compared to groups B and C (*p* < 0.05). By day 60, the highest villus to crypt ratio was observed in group D, which was significantly higher than group A (*p* < 0.05). By day 90, significant differences were observed between group C and groups A and B, with group D having significantly higher ratio compared to group C (*p* < 0.05). In summary, supplementing the feed with 0.1% concentration of *B. subtilis* and bacteriophages improves the intestinal morphology of MaGang geese.

### 3.5 Effects of dietary supplementation of *Bacillus subtilis* and bacteriophages on immune and intestinal barrier function genes

**Figure 4:**
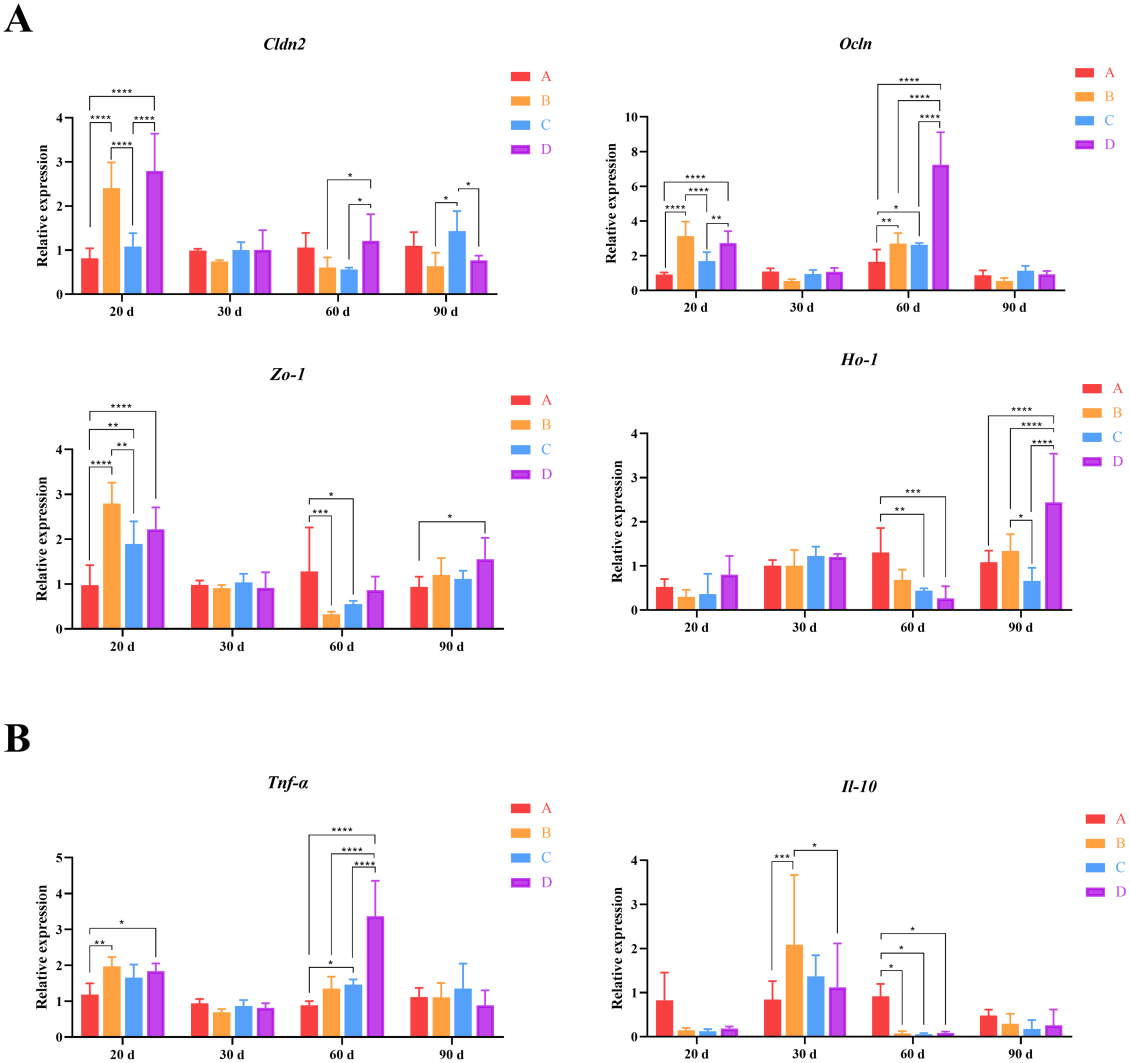
Quantitative analysis of gut-related gene expression. **(A)** Relative expression levels of intestinal tight junction proteins and oxidative stress genes. **(B)** Relative expression levels of inflammatory genes. Two-way ANOVA and multiple comparisons were conducted to assess significant interactive effects. ^*,**,***,****^ indicate means with different superscripts within the same row are significantly different (*p* < 0.05, *p* < 0.01, *p* < 0.005, and *p* < 0.001), the same below.

To investigate the effects of *B. subtilis* and bacteriophage supplementation on the expression of key genes of immunity and intestinal barrier function in Magang geese. We first examined the expression of three crucial genes, *Cldn2*, *Ocln* and *Zo-1*, which encode tight junction proteins in the intestine, maintaining intestinal morphology and protecting overall body health. By day 20, following the addition of biological agents, the expression levels of these three genes were elevated in groups B, C and D compared to group A. However, only groups B and D exhibited consistently significant differences in expression levels compared to group A (*p* < 0.001). In group C, the expression of the *Zo-1* gene was significantly higher than group A and significantly lower than group B (*p* < 0.01). Meanwhile, in group B, the expressions of the *Cldn2* and *Ocln* were significantly lower than those in groups C and D (*p* < 0.01). By day 60, a marked difference in *Ocln* expression was evident, with group D exhibiting significantly higher levels than groups A, B, and C (*p* < 0.001). Additionally, groups B and C had significant differences in *Ocln* expression when compared to group A (*p* < 0.05). Simultaneously, there were also significant differences observed in the expression of *Cldn2* and *Zo-1* expression were observed between groups B and C versus group D or group A (*p* < 0.05). However, by day 90, the situation had changed. The expression of *Cldn2* in group C was significantly higher than groups B and D (*p* < 0.05). Additionally, the expression of *Zo-1* in group D was significantly higher than group A (*p* < 0.05) (Figure 4A). These findings suggest that the combined application of *B. subtilis* and bacteriophages has a positively influences intestinal health of MaGang geese, especially on 20 days of age.

Furthermore, we also detected the expression of *Ho-1*, a gene known for its cell protective properties, which exhibits antioxidant and indirect anti-inflammatory functions. By day 60, *Ho-1* expression in group A was higher than groups B, C and D. However, only groups C and D had significant differences from group A (*p* < 0.01). By day 90, group D exhibited significantly higher expression levels compared to the other groups (*p* < 0.001). Additionally, the expression of *Ho-1* in group B was significantly higher than group C (Figure 4B). These results indicate that the addition of *B. subtilis* and bacteriophages to the diet effectively enhances the physical barrier of the intestinal tract and boosts the immune status of geese on 90 days of age.

Finally, we examined the expression of immune-related genes *Tnf-α* and *Il-10*. The results showed that *Tnf-α* was significantly differentially expressed between different groups by days 20 and 60. By day 20, *Tnf-α* expression in group A was the lowest among the four groups, with statistically significant differences compared to groups B and D (*p* < 0.05). By day 60, *Tnf-α* expression in group D was significantly higher than the other three groups (*p* < 0.001). Additionally, the expression of *Tnf-α* in group C was significantly higher than group A (*p* < 0.05). Correspondingly, the expression of *Il-10* was higher in groups B, C and D compared to group A by day 30, with group B reaching statistical significance (*p* < 0.005). By day 60, the expression level of *Il-10* in group A was much higher than that in groups B, C and D (*p* < 0.05) (Figure 4B). These findings suggest that the addition of *B. subtilis* and bacteriophages could effectively reduce the inflammatory response of goose organisms, although this effect gradually diminished over time.

### 3.6 Effects of dietary supplementation of *Bacillus subtilis* and bacteriophages on cecal microbiota

To reveal the effects of *B. subtilis* and bacteriophage addition on intestinal microorganisms of Magang geese, 16S rDNA sequencing was performed on cecum contents. The proportion of high-quality tags obtained through our sequencing process exceeded 77.24%, thus ensuring the reliability of our sequencing data (Supplementary Figure S3A). Following this, the tag sequences were clustered based on their similarity and categorized into different sequence sets, known as OTUs (Operational Taxonomic Units). Ultimately, we obtained an average of 934 OTUs per sample (Supplementary Figure S3B).

For the assessment of alpha diversity in the cecal microbiota, we meticulously calculated several indices at different stages for each group, including the observed species (Sobs), Chao1, ACE, Simpson, and Pielou indices. Specifically, by days 1, 20 and 30, our analysis revealed no significant differences among the groups for any of these diversity indices. However, by day 60, marked differences emerged. Notably, group D had the highest Sobs index, and exhibited significantly higher than group C (*p* < 0.05). Additionally, group D had the highest Chao1 index, and significantly higher than group C (*p* < 0.05). Furthermore, group D had the highest Pielou index, and significantly higher than group B (*p* < 0.05). By day 90, group D had the highest Sobs and ACE indecis, and exhibited a significant difference between groups B (*p* < 0.05). These findings indicate that the incorporation of bacteriophages and *B. subtilis* into feed of MaGang geese enhances the species richness of cecal microbiota and positively influences the gut microbiota composition (Table 4).

**Table 4:** Results of alpha diversity analysis.

Period	Groups		Sobs	Chao1	ACE	Simpson	Pielou
Day 1	A		322.83 ± 76.77	465.13 ± 41.94	494.71 ± 38.35	0.61 ± 0.12	0.27 ± 0.06
	B		337.83 ± 82.12	536.74 ± 61.78	571.23 ± 70.08	0.63 ± 0.17	0.30 ± 0.07
	C		443.83 ± 156.45	560.07 ± 103.94	589.23 ± 109.51	0.59 ± 0.13	0.29 ± 0.05
	D		486.67 ± 66.72	558.96 ± 90.09	587.56 ± 91.17	0.76 ± 0.11	0.35 ± 0.05
	Tukey’s *q* value		0.05	0.03	0.04	0.16	0.09
	*p-*value	A–B	0.994	0.973	0.992	0.994	0.792
		A–C	0.202	0.147	0.208	0.989	0.914
		A–D	0.052	0.028	0.042	0.273	0.090
	*η* ^2^		0.375	0.391	0.366	0.221	0.253
Day 20	A		775.17 ± 72.54	917.63 ± 74.83	967.91 ± 74.17	0.92 ± 0.02	0.54 ± 0.03
	B		814.00 ± 206.02	962.70 ± 219.35	1,018.29 ± 226.42	0.91 ± 0.09	0.53 ± 0.09
	C		792.00 ± 160.6	930.47 ± 181.46	986.99 ± 194.26	0.91 ± 0.05	0.53 ± 0.05
	D		768.17 ± 142.37	931.08 ± 145.39	983.53 ± 156.86	0.89 ± 0.04	0.50 ± 0.06
	Tukey’s *q* value		0.95	0.96	0.96	0.76	0.60
	*p-*value	A–B	0.971	0.964	0.957	0.931	0.973
		A–C	0.997	0.999	0.997	0.983	0.987
		A–D	1.000	0.999	0.999	0.761	0.598
	*η* ^2^		0.016	0.082	0.013	0.049	0.079
Day 30	A		1,140.67 ± 63.47	1,330.13 ± 57.76	1,425.26 ± 55.2	0.92 ± 0.05	0.56 ± 0.06
	B		1,108.33 ± 119.94	1,270.16 ± 118.46	1,365.95 ± 135.29	0.91 ± 0.04	0.54 ± 0.04
	C		1,126.00 ± 50.44	1,304.55 ± 50.4	1,397.51 ± 60.34	0.89 ± 0.03	0.52 ± 0.02
	D		1,103.00 ± 22.72	1,311.29 ± 37.33	1,398.19 ± 39.01	0.92 ± 0.04	0.57 ± 0.05
	Tukey’s *q* value		0.81	0.50	0.60	0.36	0.38
	*p-*value	A–B	0.869	0.500	0.597	0.896	0.888
		A–C	0.985	0.929	0.934	0.465	0.554
		A–D	0.810	0.969	0.938	0.998	0.990
	*η* ^2^		0.047	0.096	0.074	0.147	0.139
Day 60	A		1,142.33 ± 78.32^ab^	1,324.3 ± 92.61^ab^	1,406.57 ± 109.17	0.95 ± 0.02	0.64 ± 0.02^ab^
	B		1,246.00 ± 66.22^ab^	1,479.77 ± 68.35^a^	1,566.32 ± 76.77	0.95 ± 0.02	0.61 ± 0.03^b^
	C		1,099.00 ± 157^b^	1,299.84 ± 155.9^b^	1,384.82 ± 175.99	0.97 ± 0.01	0.67 ± 0.04^ab^
	D		1,266.17 ± 75.23^a^	1,465.96 ± 76.78^ab^	1,556.08 ± 103.41	0.96 ± 0.01	0.65 ± 0.04^a^
	Tukey’s *q* value		0.04	0.03	0.08	0.08	0.04
	*p-*value	A–B	0.313	0.077	0.139	0.936	0.628
		A–C	0.879	0.977	0.989	0.238	0.323
		A–D	0.180	0.119	0.180	0.694	0.938
	*η* ^2^		0.364	0.420	0.358	0.278	0.313
Day 90	A		1,116.83 ± 146.59^ab^	1,314.92 ± 172.93	1,404.06 ± 174.58^ab^	0.95 ± 0.03	0.6 ± 0.06
	B		1,106.00 ± 168.92^b^	1,290.54 ± 164.85	1,360.00 ± 178.38^b^	0.96 ± 0.04	0.65 ± 0.06
	C		1,139.17 ± 192.97^ab^	1,311.33 ± 181.93	1,396.83 ± 203.19^ab^	0.97 ± 0.02	0.68 ± 0.04
	D		1,362.00 ± 86.27^a^	1,541.72 ± 97.61	1,638.69 ± 102.22^a^	0.97 ± 0.01	0.66 ± 0.03
	Tukey’s *q* value		0.04	0.05	0.04	0.32	0.05
	*p-*value	A–B	0.999	0.993	0.968	0.923	0.272
		A–C	0.994	1.000	1.000	0.327	0.054
		A–D	0.054	0.093	0.108	0.381	0.166
	*η* ^2^		0.359	0.337	0.339	0.174	0.299

^a,b^Means in the same row with different superscripts (a, b) differ significantly between two columns (*p* < 0.05).

**Figure 5:**
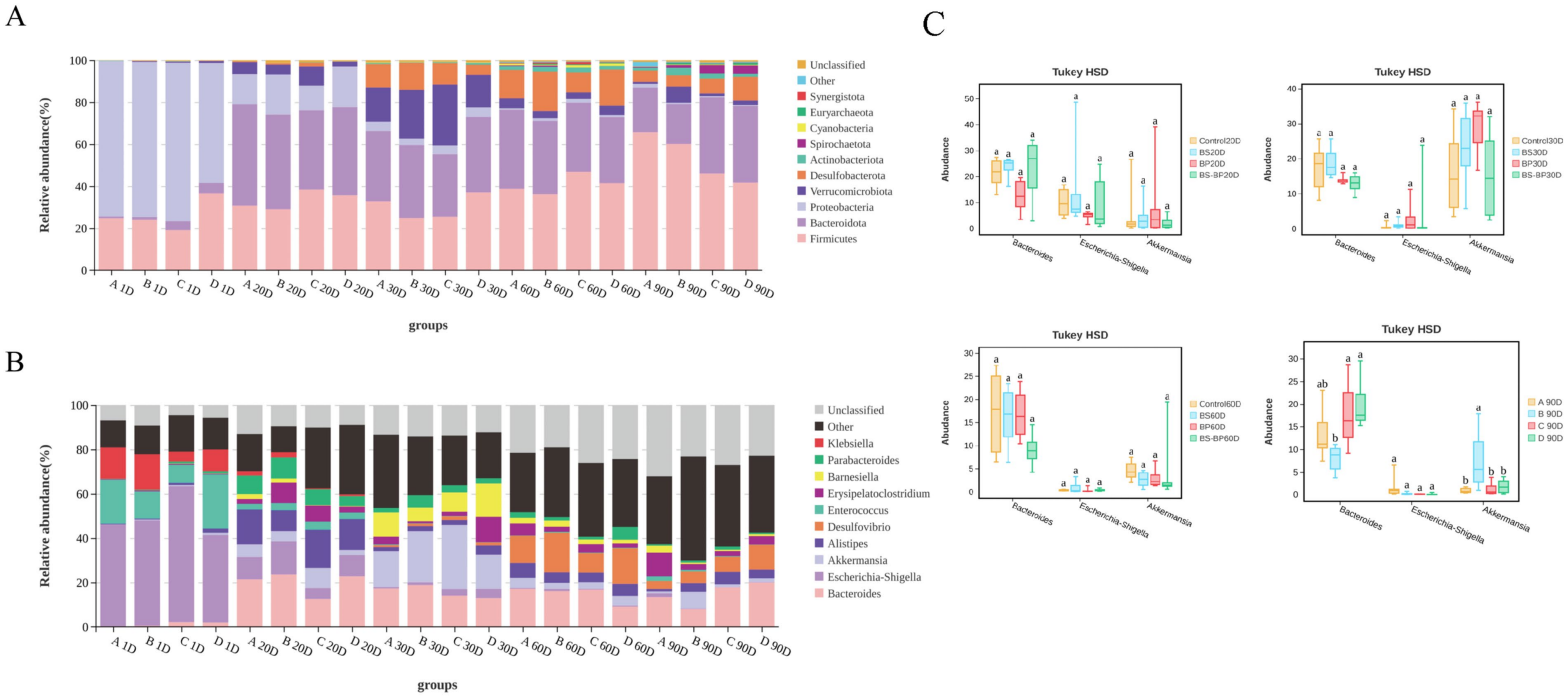
Relative abundance of cecal microbiota in geese. **(A)** Relative abundance of cecal microbiota at the phylum level in geese. **(B)** Relative abundance of cecal microbiota at the genus level in geese; **(C)** Comparative analysis of the top three most abundant genera in cecal microbiota composition at the genus level among geese.

Regarding the species abundance of cecal microbiota, our analysis revealed that the predominant bacterial phyla and genera varied over time. By day 1, the predominant phyla were *Protebacteria*, *Firmicutes, Bacteroidota*, with *Escherichia*, *Enterococcus* and *Klebsiella* being the most abundant genera in the intestinal flora. By day 20, the predominant phyla had changed. The relative abundance of *Proteobacteria* decreased markedly, whereas the relative abundances of *Firmicutes* and *Verrucomicrobiota* increased significantly. The main bacterial genera at this point were *Bacteroides*, *Alistipes* and *Escherichia*. By day 30, the predominant phyla continued to vary. The relative abundance of *Proteobacteria* continued to decrease, whereas the relative abundances of *Desulfobacterota* increased significantly. The dominant bacterial genera at this stage were *Bacteroides*, *Akkermansia* and *Barnesia*. By day 60, the predominant phyla were *Firmicutes, Bacteroidota*, and *Desulfobacterota*. The main genera were *Bacteroides*, *Desulfovibrio* and *Alistipes*. Finally, by day 90, the predominant phyla remained consistent with day 60, and *Bacteroides* and *Desulfovibrio* were the predominant bacteria genera (Figures 5A,B). Notably, when examining the top three genus (*Bacteroides*, *Escherichia-Shigella* and *Alistipes*) across different experimental groups at the same time point, significant differences were found by day 90 (Figure 5C).

**Figure 6:**
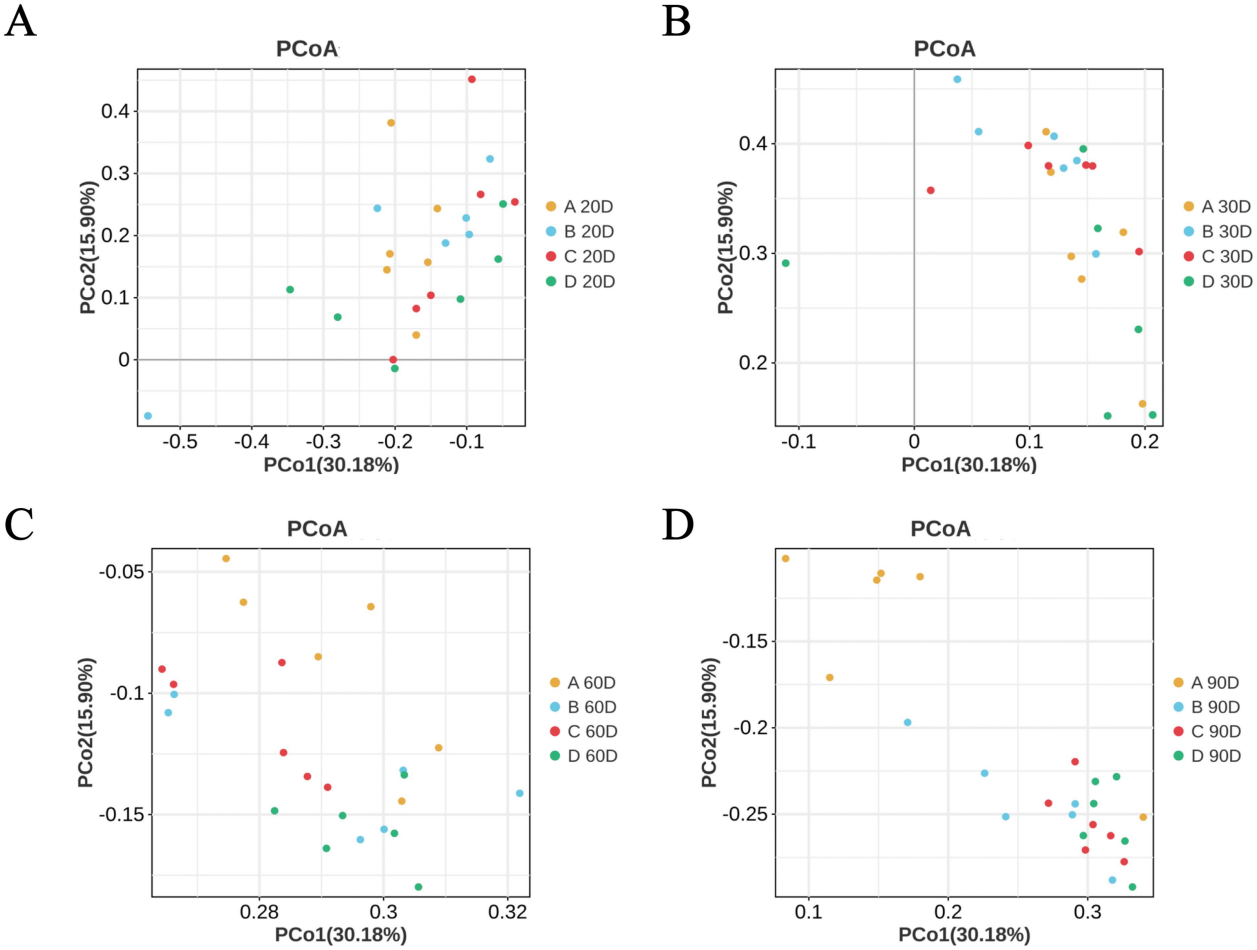
Principal Coordinate Analysis (PCoA) analysis based on unweighted UniFrac distances. **(A)** PCoA analysis at day 20. **(B)** PCoA analysis at day 30. **(C)** PCoA analysis at day 60. **(D)** PCoA analysis at day 90.

The beta diversity of cecal microbiota in MaGang geese exhibited no significant differences in the composition by days 10 and 30 (Figures 6A,B). However, as the time progressed, notable changes emerged. By day 60, the composition of cecal microorganisms began to gradually, with the different treatment groups exhibiting alterations in a consistent direction compared to the control group (Figure 6C). By day 90, the different treatment groups were markedly distinct from the control group, yet the microbial composition of the cecal contents within the various treatment groups remained highly similar (Figure 6D). These findings indicate that the addition of microbial agents to the diet of MaGang geese can effectively modify and improve the intestinal microbiota of these birds.

### 3.7 Correlation of gut microbiota with growth performance, intestinal immune function and barrier-related parameters

To investigate the relationship between various environmental factors and cecal microbiota, we conducted a Pearson correlation analysis focusing on the top 10 significant microbiota and their relationships with related gene expression levels, growth performance, intestinal barrier, endotoxins, and environmental bacterial counts.

**Figure 7:**
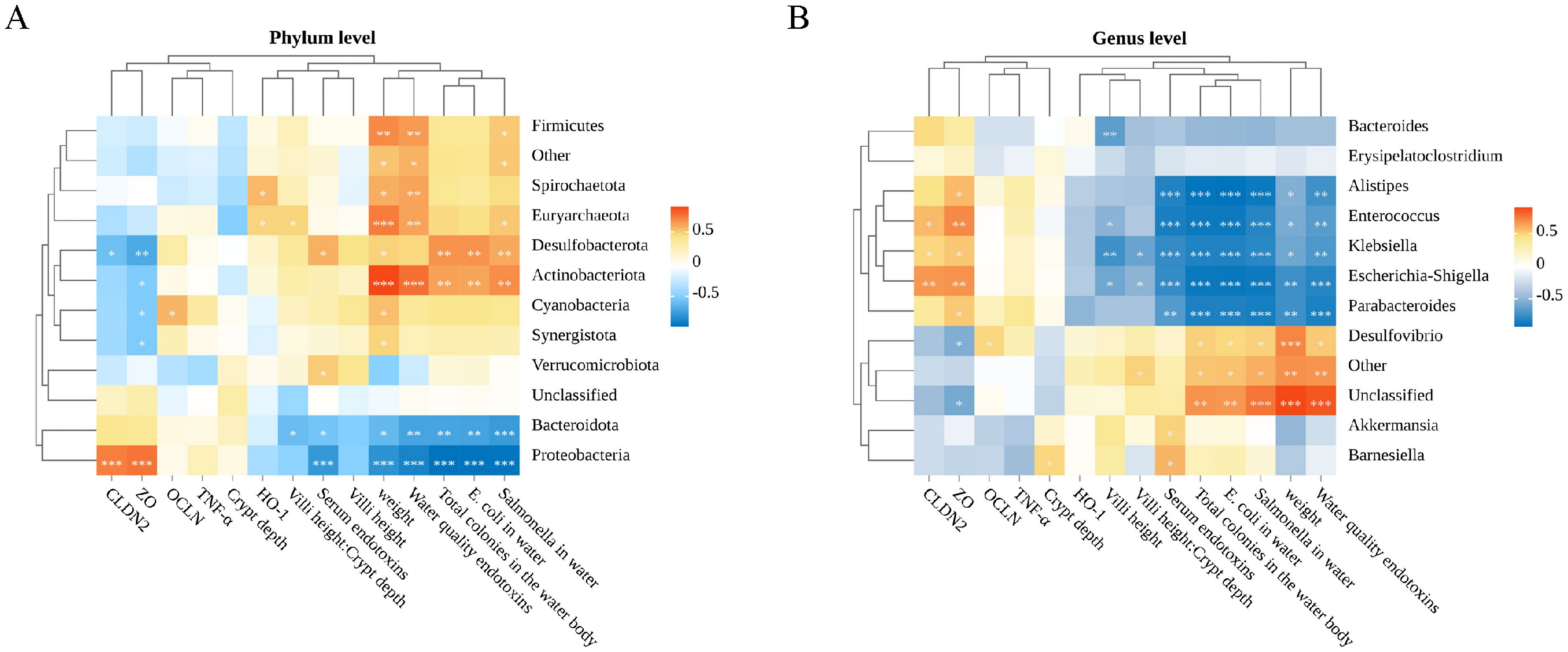
Spearman correlation analysis. **(A)** Spearman correlation analysis of water toxins, serum endotoxins, growth performance, water bacterial colonies, relative expression of intestinal genes, intestinal histology data, and gut microbiota (phylum level). **(B)** Spearman correlation analysis of water toxins, serum endotoxins, growth performance, water bacterial colonies, relative expression of intestinal genes, intestinal histology data, and gut microbiota (genus level).

As illustrated in Figure 7A, the top 10 bacteria phyla exhibited correlations with water toxins, serum endotoxins, growth performance, water colonies, relative expression of intestinal genes and intestinal section data. Specifically, *Firmicutes*, *Spirochaetota*, *Euryarchaeota*, *Desulfobacterota*, *Actinobacteriota*, *Cyanobacteria* and *Synergistota* demonstrated significant positive correlations with geese weight, endotoxins and bacterial colonies in water quality (*p* < 0.05). Conversely, these phyla showed significant negative correlations with the expression of *Cldn2* and *Zo-1,* which are integral proteins of the intestinal tight junction. In contrast, *Bacteroidota* and *Proteobacteria* exhibited opposite correlations compared to the aforementioned microbial communities (*p* < 0.05).

We further delved into the analysis at the genus level. As depicted in Figure 7B, *Gram negative bacteria Desulfovibrio, Akkermansia and Barnesiella*, were positively correlated with the number of *bacteria* and *endotoxins* levels in the environment (*p* < 0.05). Notably, these genera did not exhibit strong negative correlations with gut related genes or inflammatory factors. Conversely, *Escherichia-Shigella*, *Alistipes*, *Enterococcus*, *Parabacteroides* and *Klebsiella*, demonstrated strong positive correlations with intestinal tight junction proteins but showed strong negative correlation with the number of *bacteria* and *endotoxins* levels in the environment (*p* < 0.05). It is worth noting that these microbial communities did not exhibit significant correlations with oxidative stress (*p* > 0.05).

## 4 Discussion

The interplay between water quality management and immune health in waterfowl production systems presents a critical challenge, particularly in intensive farming practices. Conventional approaches to maintaining bathing pool hygiene often lead to biological pollution and disease transmission, as observed in high-density semi-dry farming systems where water contamination exacerbates antibiotic overuse and compromises animal welfare (4, 7). This underscores the urgent need for sustainable alternatives to antibiotics, with probiotics and bacteriophages emerging as promising candidates for mitigating ecological and public health risks.

Probiotics, for instance, exhibit diverse beneficial effects in poultry, including enhanced production performance, disease prevention, and improved host health (18). Among these, *B. subtilis* microecological agents have emerged as promising antibiotic alternatives in livestock and poultry industries. Studies demonstrate that *B. subtilis* supplementation promotes nutrient absorption, optimizes feed conversion efficiency, and stimulates growth in animals. For example, Drinking Water supplementation with *B. subtilis* PS-216 significantly increases weight gain in chickens (19), while analogous benefits were observed in swine (20).

In addition to probiotics, bacteriophages, as naturally occurring antibacterial agents, offer another innovative solution. These viruses regulate bacterial populations through targeted lysis and exhibit activity against both Gram-negative and Gram-positive bacteria, including multidrug-resistant pathogens (13, 21). Furthermore, bacteriophages have demonstrated efficacy in biocontrol applications, such as reducing Salmonella contamination in poultry products to enhance food safety (22–26). Domestic studies highlight bacteriophages as sustainable alternatives to antibiotics, with evidence of growth-promoting effects in broilers (7). Increasing evidence supports their environmentally benign profile and potential utility in feed additives or disease prevention strategies (18, 20, 27).

Given the potential of these agents, we propose integrating *B. subtilis* and bacteriophages into the dietary regimen of Magang geese. Building on empirical findings from controlled water sample experiments, this dual approach aims to evaluate their synergistic effects on growth performance, intestinal health, and gut microbiota composition, as well as assess their capacity to reduce endotoxin levels in both geese and cultured water, and lower total bacteria on PCA plates counts in farming water. By elucidating the multifunctional benefits of these agents, this study seeks to advance sustainable practices in waterfowl production.

In the present study, dietary supplementation with *B. subtilis* and bacteriophages significantly enhanced the growth performance of Magang geese, as evidenced by higher average body weight and lower feed-to-gain ratio in supplemented groups compared to the control. These improvements align with previous studies demonstrating the efficacy of *B. subtilis*-fermented products in improving the growth performance and modulate the gut microflora composition of broilers under immune stress (28). The synergistic enhancements in intestinal health, including structural reinforcement through increased jejunal villus height, are consistent with the role of *B. subtilis* in promoting intestinal epithelial development, as reported by Kim et al. (29). Furthermore, the upregulated expression of tight junction protein genes (*Cldn2*, *Ocln*, *Zo-1*) collectively expanded nutrient absorption capacity while reducing intestinal permeability. These observations align with the findings of Chen et al. (30), who reported that *B. subtilis* supplementation strengthens intestinal barrier function via tight junction protein regulation in Laying Hens.

The concurrent up-regulation of tight junction protein genes (*Cldn2*, *Ocln*, *Zo-1*) underscores a reinforced intestinal barrier, which likely attenuated systemic endotoxin (LPS) leakage. The reduction in endotoxin levels in our findings can be attributed to two main mechanisms. On the one hand, the phage’s capacity to selectively lyse Gram-negative pathogens, such as *E. coli* and *Salmonella*, directly diminishes the source of LPS. On the other hand, *B. subtilis* produces antimicrobial metabolites, including surface-active substances and bacteriocins, which inhibit pathogen colonization and enrich beneficial taxa, such as *Firmicutes* and *Bacteroidetes*. This shift in microbial composition further reduces the dominance of Gram-negative bacteria, consequently decreasing endotoxin production. These observations are consistent with the decreased counts of *E. coli* and *Salmonella* detected in the cultured water of our supplemented groups compared to the control group. Our findings align with previous studies highlighting the efficacy of bacteriophages in lysing pathogenic *E. coli* and *Salmonella*, thereby directly curtailing LPS release into aquatic environments. Similarly, *B. subtilis*-mediated production of antimicrobial metabolites, such as surfactants and bacteriocins, corroborates earlier work showing its ability to inhibit pathogen colonization and promote beneficial gut microbiota (31).

To further investigate the impact on intestinal microbiota, 16S rDNA sequencing of cecum contents was utilized. Our findings reveal that the addition of microbial agents to the diet of MaGang geese can effectively modify and improve the intestinal microbiota. Specifically, group D exhibiting the highest microbial diversity and sustained divergence. Furthermore, the supplemented groups showed increased abundances of *Firmicutes* and *Bacteroidota*, phyla associated with short-chain fatty acid (SCFA) production, as well as reduced pathogenic *Proteobacteria*, such as *Escherichia-Shigella* and *Salmonella*. The enrichment of *Bacteroidota* (e.g., *Bacteroides*) likely up-regulated tight junction proteins via metabolite signaling, while SCFAs fortified the mucus layer, establishing a reinforcing loop between barrier integrity and microbiota balance. This mechanism mirrors findings in laying hens, where *B. subtilis* supplementation mitigated gut inflammation and oxidative stress via microbiota modulation (32). The dual intervention further enriched *Firmicutes* and *Bacteroidota*, which are phyla linked to short-chain fatty acid (SCFA) biosynthesis, while concurrently suppressing pathogenic *Proteobacteria* such as *Escherichia-Shigella*. Such microbial shifts align with mouse model where SCFA-producing taxa enhanced barrier function and anti-inflammatory responses (33), suggesting a conserved mechanism across species.

Through the correlation analysis of environmental factors and intestinal microbiota, we also determined that the expression of intestinal tight junction protein is inextricably linked to changes in microbiota, which has been demonstrated by other reports (34, 35). Our findings showed that endotoxin levels could be further reduced by reducing the number of *E. coli, Salmonella* and the total number of colonies in the water body, not only the endotoxin level in the water body but also in the serum, while reducing the expression level of the inflammatory factor (17), which proves that the gut as a physical barrier effectively enhancing the host’s immune system. These findings were also proved by the results of increased gut microbial biota diversity. At the same time, we also focus on the increase in the expression of some antioxidant genes (like *HO-1*), which is similar to the findings of Zhang et al., showing that *B. subtilis* and bacteriophages can enhance the intestinal barrier’s immunity by increasing antioxidant capacity (36), thus protecting the breeding health of MaGang goose.

The integration of *B. subtilis* and bacteriophages addresses critical gaps in antibiotic-free waterfowl production. Although no positive control for the antibiotic group was conducted in our experimental design, the previous studies showed that chickens fed with 1 and 1.5 g of BP/kg had a taller and more regular villus than the control and colistin treatments (17). Grozina et al. conducted a 36-day feeding trial on Cobb 500 broilers and observed that chickens supplemented with either probiotics or antibiotics exhibited greater gut microbial richness compared to the negative control group. Furthermore, both the probiotic and antibiotic groups demonstrated higher abundances of beneficial bacterial populations and lower levels of pathogenic bacteria in the intestinal tract than the control group (37). These findings suggest that probiotics and antibiotics share similar efficacy in modulating gut microbiota composition. According to the results of Ji et al., the addition of *B. subtilis* to the diet can achieve the same antioxidant and intestinal microbiota improvement effects as antibiotics (38), and according to the study of Bilal et al., the addition of *B. subtilis* to the diet has a higher expression of intestinal tight junction protein in chickens than antibiotics and better inhibition of validation (34). Kalani et al. conducted a comparative study on the effects of antibiotics, probiotics, and prebiotics in chickens. The results demonstrated that both antibiotics and probiotics significantly reduced serum levels of low-density lipoprotein (LDL), triglycerides, and cholesterol, while effectively alleviating Salmonella infection. Notably, probiotics exhibited superior efficacy compared to antibiotics (39). These findings further substantiate the potential of probiotics as a viable alternative to antibiotics in poultry production. By concurrently targeting intestinal health and environmental microbiota, this strategy holds the potential to mitigate antibiotic resistance gene transfer and enhance production efficiency. However, further research is warranted to validate the long-term safety, phage-host specificity, and ecological impacts of this approach under field conditions. Moreover, elucidating the molecular interactions between *B. subtilis* metabolites and phage dynamics could refine their combined application.

Ultimately, the implementation of *B. subtilis* and bacteriophages as dual microecological agents in Magang geese farming systems represents a novel strategy that reconciles growth performance, intestinal health, and environmental sustainability. Our findings demonstrate that this synergistic approach not only augments host physiology but also mitigates ecological risks associated with conventional antibiotic-dependent practices, thereby advocating for a paradigm shift toward One Health-aligned waterfowl production.

## 5 Conclusion

**Figure 8:**
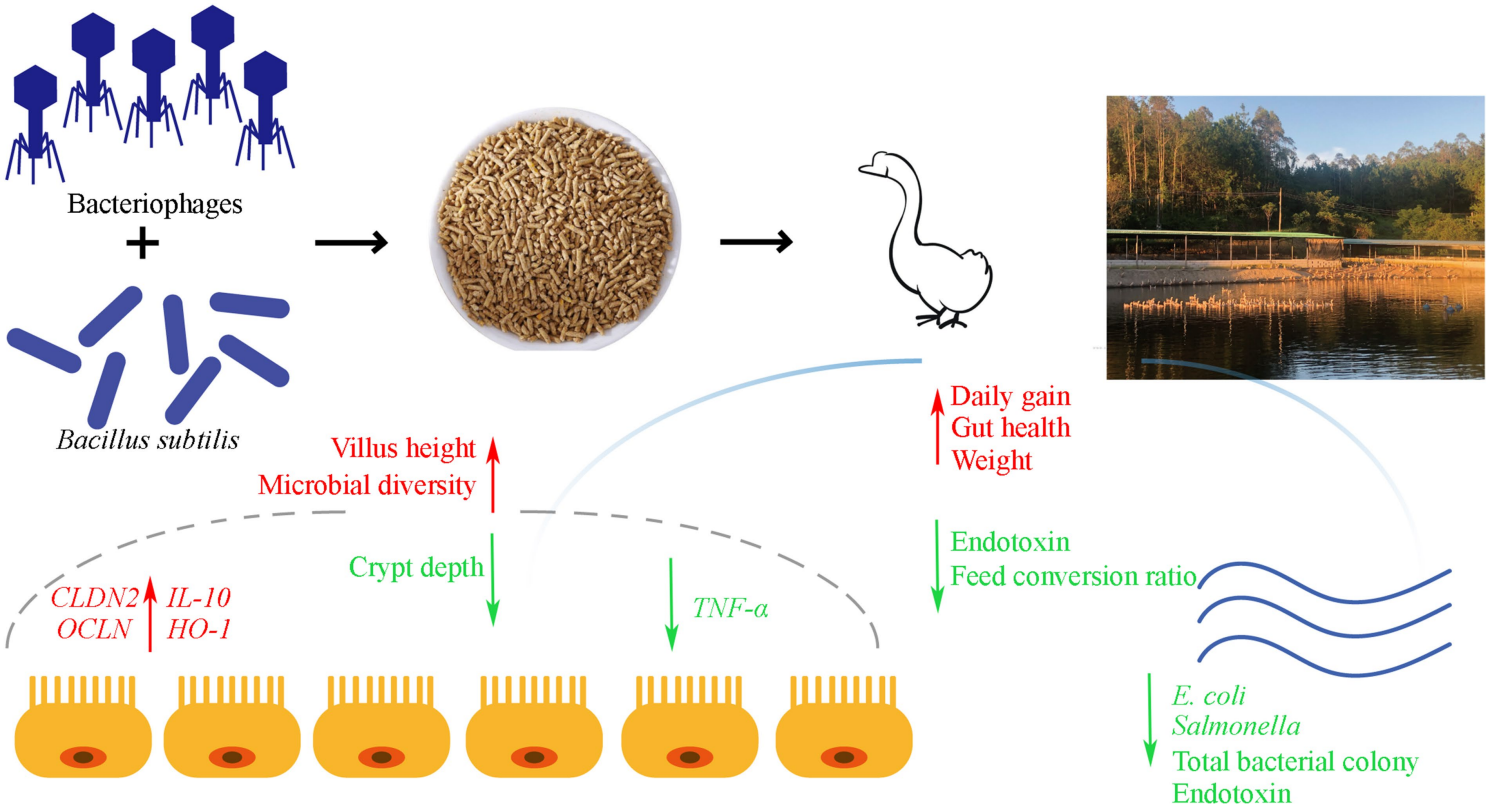
Graphical summary of dietary supplementation with *Bacillus subtilis* and bacteriophages on the production performance and intestinal health of MaGang geese.

In summary, our study revealed that the concurrent administration of *B. subtilis* and bacteriophages significantly enhances the intestinal barrier function in geese. This enhancement is evidenced by increased expression of tight junction protein genes, reduced serum endotoxin levels, and an improved ratio of intestinal villus height to crypt depth. Furthermore, dietary supplementation with *B. subtilis* and bacteriophages effectively controls the proliferation of harmful microbial communities in the gastrointestinal environment, thereby promoting overall growth performance in geese (Figure 8).

## Data Availability

The datasets presented in this study can be found in online repositories. The names of the repository/repositories and accession number(s) can be found in the article/Supplementary material.

## Footnotes

^1^ http://www.omicshare.com/tools
