# Supplementary material for: Effects of dietary supplementation with Bacillus subtilis and bacteriophage on growth performance, intestinal morphology and microbiota structure in 0–90 d MaGang geese
Source: Front Nutr. 2025 Apr 2;12:1537724. doi: 10.3389/fnut.2025.1537724 (PMC11999834; doi:10.3389/fnut.2025.1537724)
Supplement: Supplementary file 1 [file Data_Sheet_1.docx]

Supplementary Material

# Supplementary Data

The following are available online in the NCBI primary data archive with accession number ([PRJNA1154467](https://www.ncbi.nlm.nih.gov/bioproject/PRJNA1154467)).

# Supplementary Figures and Tables

**Supporting information captions:**

**Table S1. Composition and nutrient levels of experimental grains**

| Nutrient levels | Content (%) |
| --- | --- |
| Crude protein | 12.0 |
| Fiber | 12.0 |
| Crude ash | 12.0 |
| Calcium | 0.5-1.2 |
| Total phosphorus | 0.3-1.2 |
| Sodium chloride | 0.25-0.8 |
| Moisture | 13.5 |
| Lysine | 0.5 |

**Table S2. Primer sequences for RT-qPCR**

| Primer name | Forwad （5'-3'） | Reverse（3'-5'） | Annealing temperature （℃） |
| --- | --- | --- | --- |
| *β-actin* | ATGTCGCCCTGGATTTCG | CACAGGACTCCATACCCAAGAA | 60 |
| *Cldn2* | ACCCCGTTACAGTTCAGACG | CTGGTTACAGGAAGGGCAAC | 60 |
| *Ocln* | CAGGATGTGGCAGAGGAATACAA | CCTTGTCGTAGTCGCTCACCAT | 60 |
| *Zo-1* | GCCTGCCACATTGTGACCC | ACGTAAGGTCCATCTCAGTTTCA | 60 |
| *Ho-1* | ATGCCTACACTCGCTATCTG | GCAAGGTCCATCTCAAGG | 60 |
| *Tnf-α* | ATGAACCCTCCTCCGTACAC | AGAGGCCACCACATGATAGC | 60 |
| *Il-10* | ATCATGACATGGACCCGGTA | ATTGCTCCATGACAGTTGCT | 60 |

**2.2 Supplementary Figures**

**
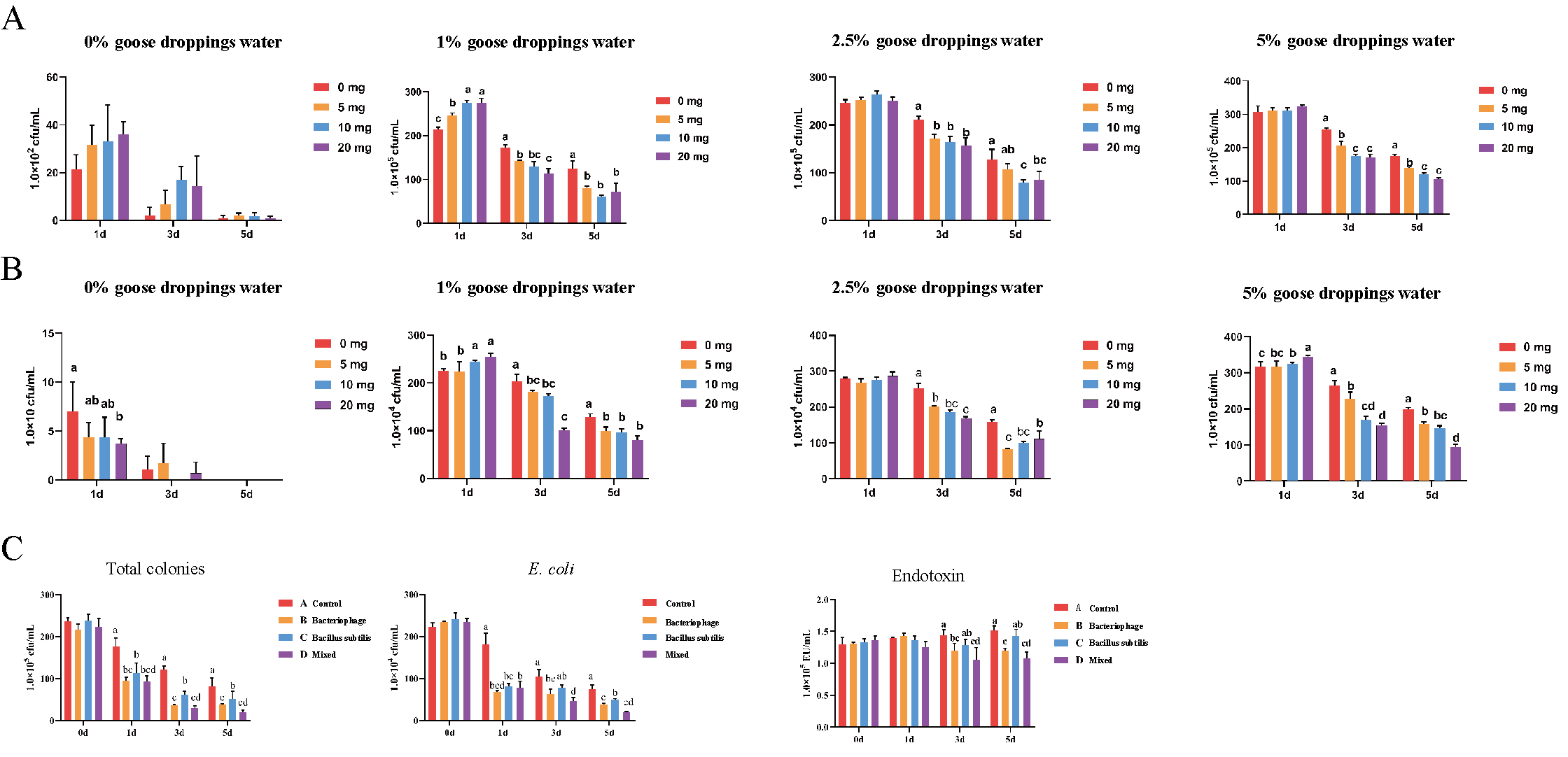
**

**Supplementary Figure 1.**  **Purification of goose manure water by different concentrations of *Bacillus subtilis* and bacteriophages.** **A**:Effect of different doses of *Bacillus subtilis* on total colonies in goose manure water at different concentrations; **B**:Effect of different doses of *Bacillus subtilis* on *E. coli* in different concentrations of goose manure water; **C**:Effect of the use of *Bacillus subtilis* or bacteriophage in 5% goose faecal water on total colonies, *E. coli* and endotoxins in water.


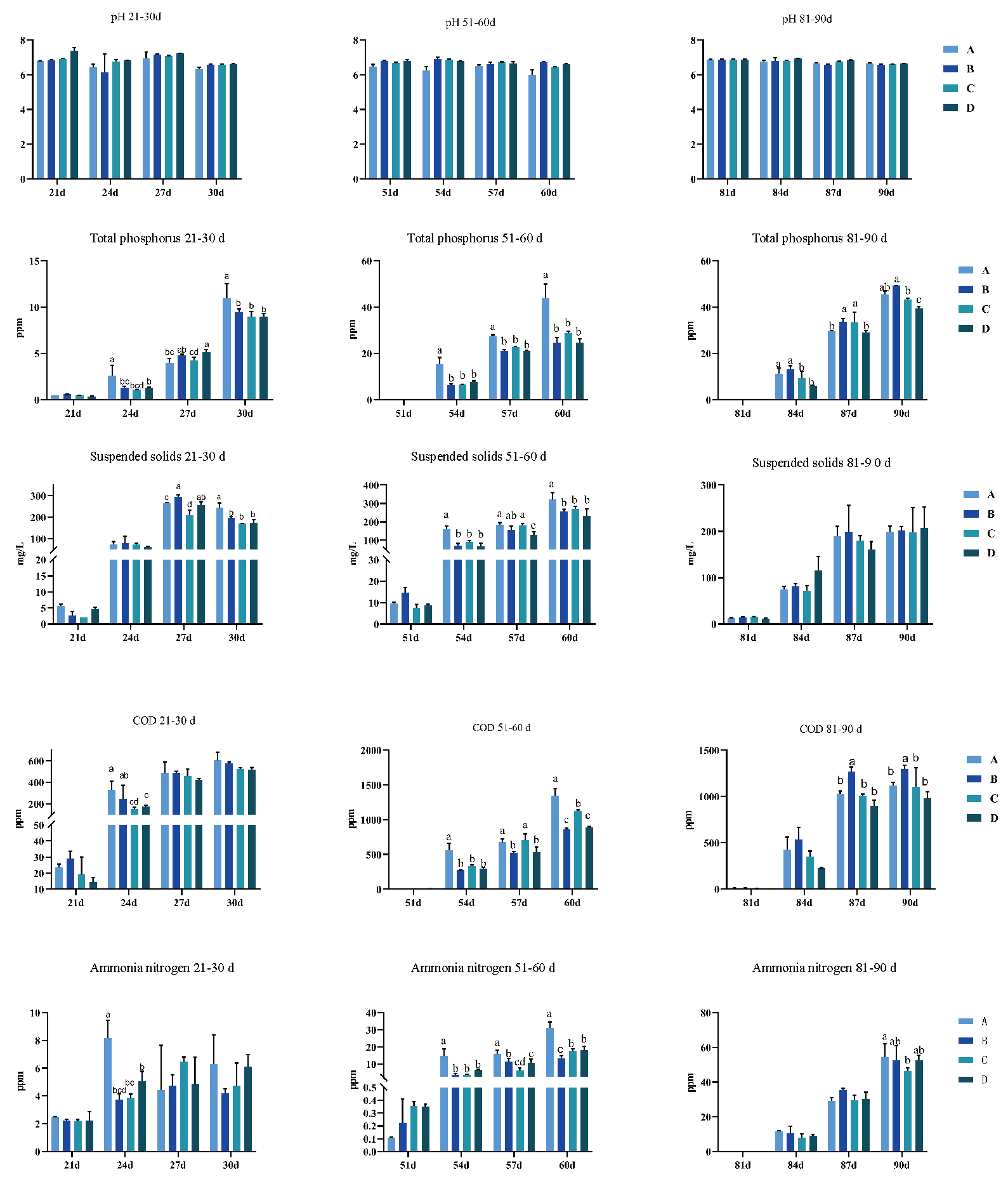


**Supplementary Figure 2.**  **Effects of Bacillus subtilis and bacteriophage on the physicochemical properties of water quality in goose baths at different stages.**


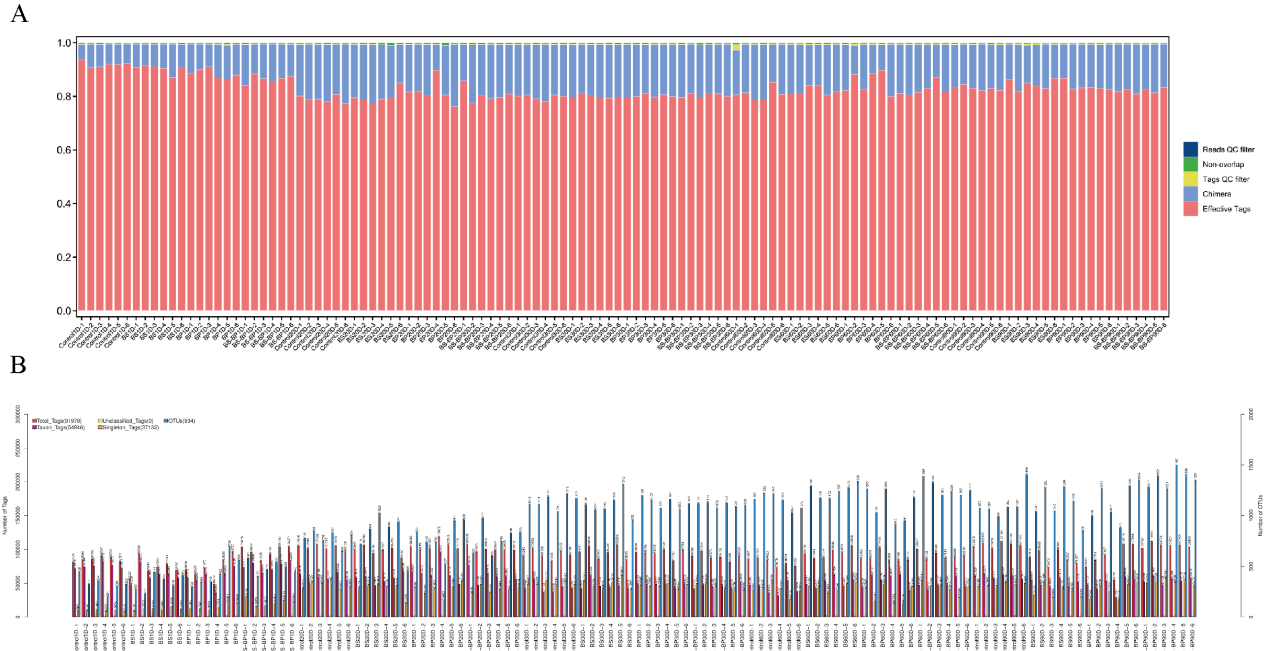


**Supplementary Figure 3.**  **16s DNA sequencing quality control.** **A**:Data preprocessing distribution (%). **B**:Statistics of the number of OTUs and Tags of different samples.
